# Circ-SHPRH in human cancers: a systematic review and meta-analysis

**DOI:** 10.3389/fcell.2023.1182900

**Published:** 2023-05-25

**Authors:** Hong Xiong, Gaozhen Huang, Yi Zhu, Ruiqi Chen, Ling Zuo, Hongwei Liu

**Affiliations:** ^1^ Laboratory of Urology, Affiliated Hospital of Guangdong Medical University, Zhanjiang, China; ^2^ Department of Traditional Chinese Medicine, The Second Affiliated Hospital of Guangdong Medical University, Zhanjiang, China

**Keywords:** Circ-SHPRH, hsa_circ_0001649, cancer, biomarker, circRNA

## Abstract

Circular RNA (circRNA) molecules are noncoding RNAs with ring-like structures formed by covalent bonds and are characterized by no 5*′*caps or *3′*polyadenylated tails. Increasing evidence shows that circRNAs may play an important role in tumorigenesis and cancer metastasis. Circ-SHPRH originates from exons 26–29 of the *SHPRH* gene, and it is closely associated with human cancers. We searched PubMed, Web of Science, and Embase databases for relevant literatures until 24 December 2022. Eighteen research papers were included in this review, and 11 papers were selected for meta-analysis after screening. Three eligible published studies about circ-SHPRH were enrolled based on their tumor diagnosis aspect, 7 eligible published studies were related to overall survival (OS), and 3 eligible published studies were related to tumor grade. Many studies have shown that circ-SHPRH acts as a miRNA sponge or encodes a protein to regulate downstream genes or signal pathways, and exerts specific biological functions that affect the proliferation, invasion, and apoptosis of cancer cells. Meta-analysis showed that patients with high expression of circ-SHPRH had better OS (HR = 0.53, 95% CI 0.38–0.74, *p*-value <0.05) and lower TNM stage (HR = 0.33, 95% CI 0.18–0.62, *p*-value = 0.001). In addition, circ-SHPRH has potential diagnostic value (AUC = 0.8357). This review will help enrich our understanding of the role and mechanism of circ-SHPRH in human cancers. Circ-SHPRH has the potential to be a novel diagnostic and prognostic biomarker for various solid cancers.

## 1 Introduction

**FIGURE 1:**
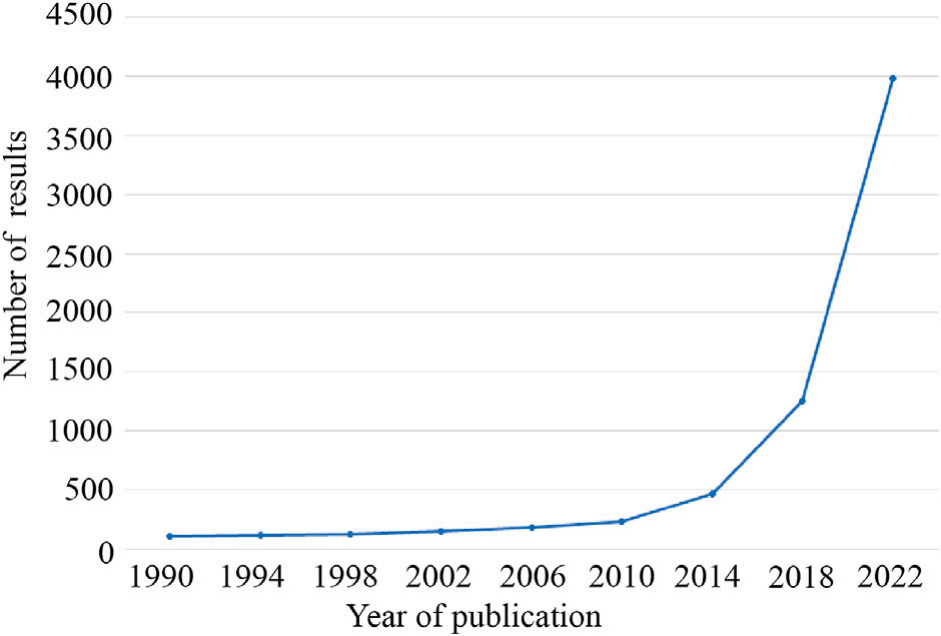
The increasing trend of the number of studies on circRNAs. A search was performed on PubMed using the keywords “circRNA” or “circular RNA”.

**FIGURE 2:**
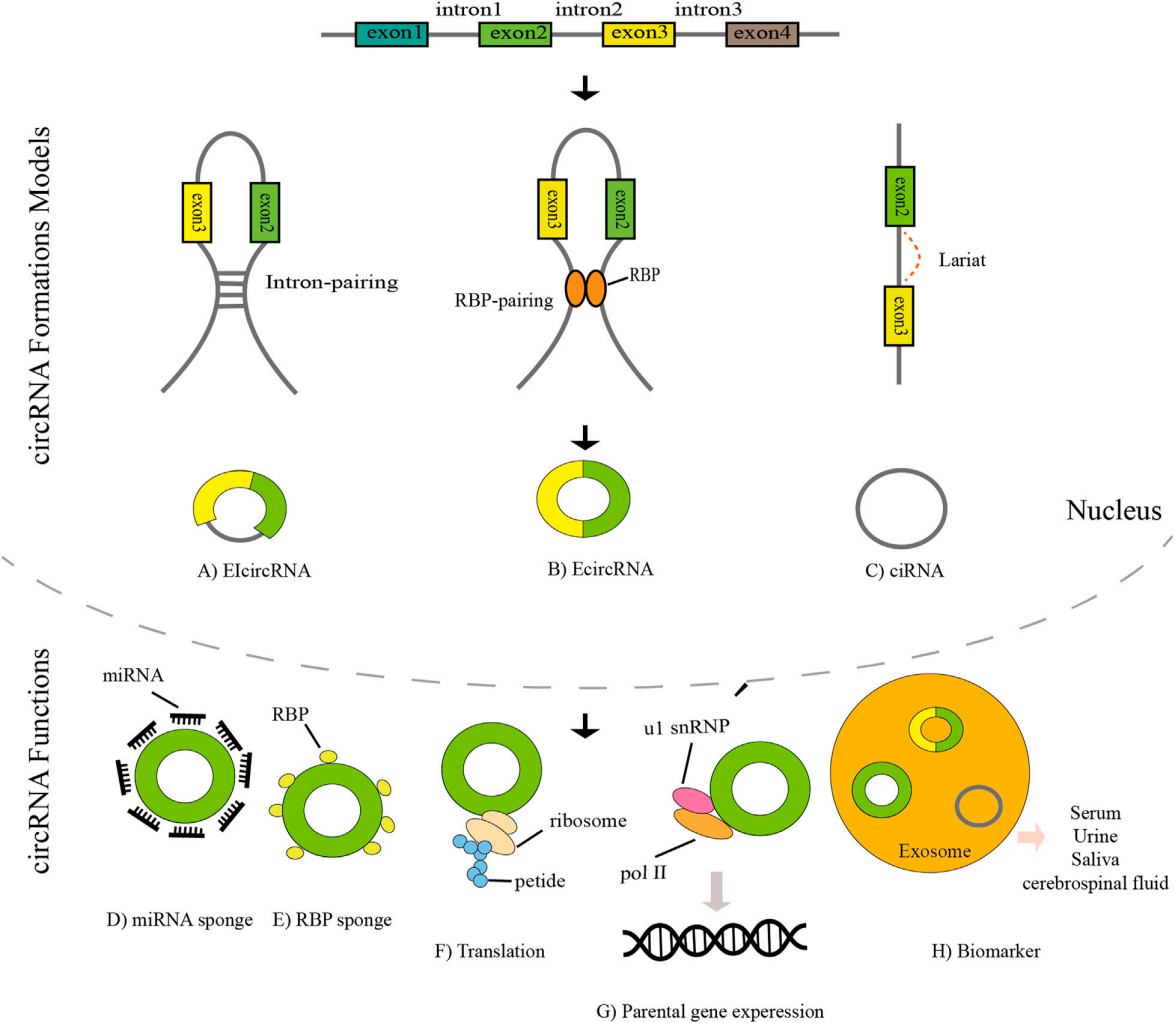
The biogenesis, mechanism, and clinical significance of circRNAs. **(A)** Exon–intron circRNAs (EIcircRNAs) contain both exons and introns. **(B)** Exonic RNAs (EcircRNAs) are generated from exons. **(C)** CircRNAs are formed by introns. **(D)** CircRNAs can act as miRNA sponges. **(E)** CircRNA binds to RNA-binding protein (RBP) to form an RNA–protein complex (RPC). **(F)** CircRNAs can directly recruit ribosomes and be translated. **(G)** CircRNAs can combine with U1 snRNP and interact with Pol II to enhance parental gene expression. **(H)** CircRNAs can act as biomarkers.

In recent years, the worldwide incidence of cancer has been high, which has seriously affected human health (Wang M. et al., 2018). Some studies estimate that the number of cancer patients worldwide is expected to reach 28.4 million by 2040 (Sung et al., 2021). In 1976, Sanger et al. first discovered circular RNAs in plant viruses (Sanger et al., 1976). Due to the limitations of technology and understanding at the time circRNA was first discovered, it was considered an RNA molecule formed by erroneous splicing, and it did not attract attention. In particular, the development of high-throughput sequencing technology has greatly aided in circRNA research, and circRNAs are being increasingly identified. In recent years, circRNA has become a research hotspot (Figure 1). The tissue-specific and developmental-stage-specific expression patterns of circRNA are similar to those of the corresponding linear mRNA, but the expression levels of circRNAs are more than 10-fold higher than those of the corresponding linear mRNA (Memczak et al., 2013). CircRNA also has the characteristic of an evolutionarily conserved sequence. The circular structure of circRNA helps prevent RNA exonuclease degradation. In addition, because of the characteristic of a closed loop structure, the half-life of circular RNA is longer than that of linear RNA in most eukaryotes, and circRNA appears to be more stable than linear RNA (Suzuki and Tsukahara, 2014; Bahn et al., 2015). CircRNAs are derived from linear pre-mRNAs transcribed by RNA polymerase II(Ashwal-Fluss et al., 2014; Zhang et al., 2014; Starke et al., 2015). There are three mechanisms of circRNA formation: intron pairing-driven circularization, RNA-binding protein-mediated circularization, and lariat-driven circularization (Holdt et al., 2018). Acting as a miRNA sponge is one of the most frequently studied features of circRNAs. CircRNAs contain multiple miRNA response elements (MREs) and are competitive endogenous RNAs (ceRNAs) (Hansen et al., 2013). CircRNAs can also interact with RNA-binding proteins, regulate alternative gene transcription, and act as a biomarker. In addition, a very small number of circRNAs have the function of translation (Figure 2) (Zlotorynski, 2015; Du et al., 2017; Legnini et al., 2017; Weng et al., 2017). In recent years, an increasing number of scholars have found that circular RNAs play a significant role in the proliferation and migration of tumor cells and other pathophysiological processe (Li et al., 2018; Yang et al., 2018).

**FIGURE 3:**
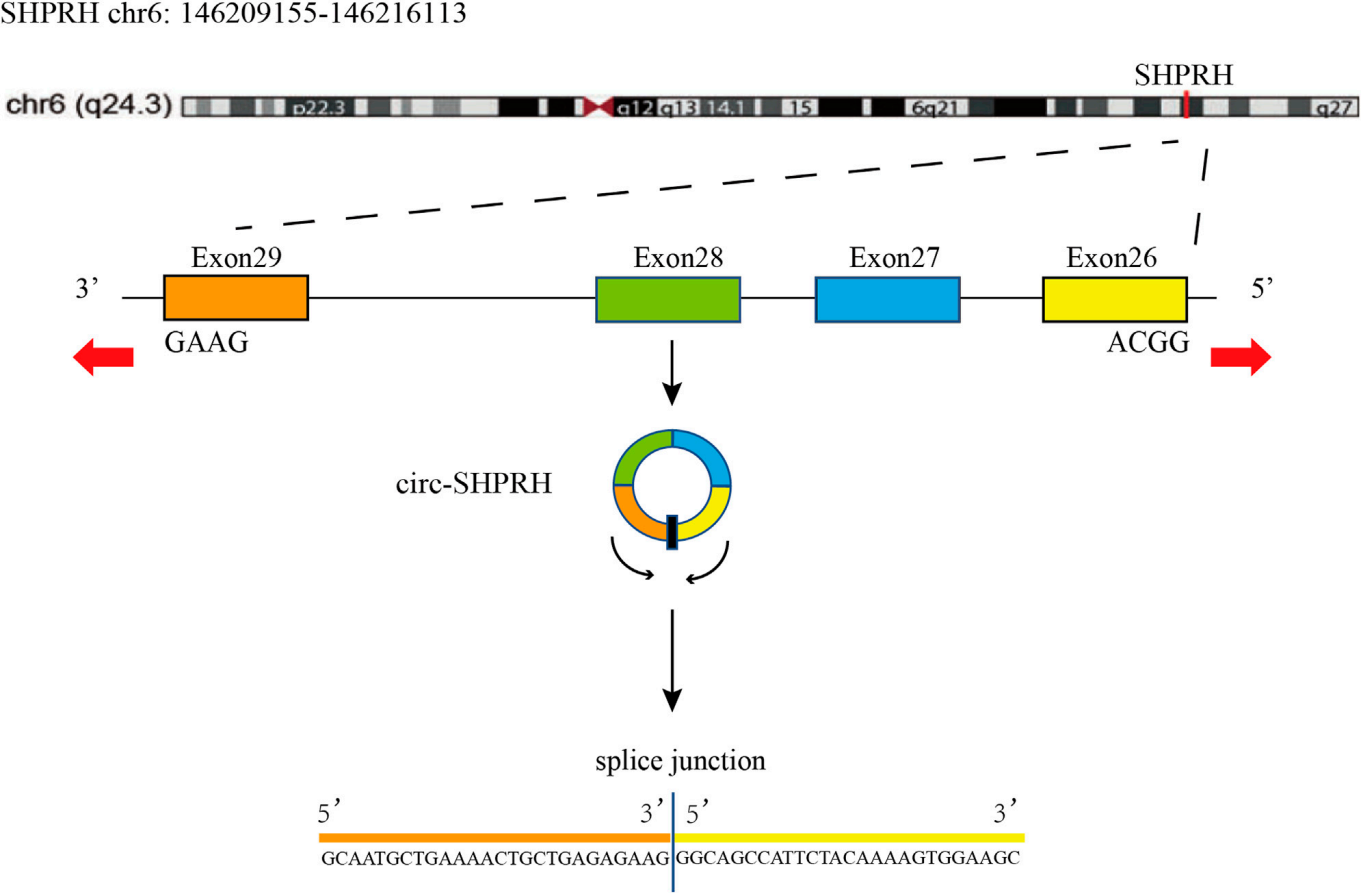
Schematic illustration of the formation of circ-SHPRH.

Circ-SHPRH (circbase ID: hsa_circ_0001649), produced by the SNF2 histone linker PHD RING helicase (*SHPRH*) gene, is located at chr6: 146209155–146216113 (Sood et al., 2003). Circ-SHPRH is formed by the back splicing of exons 26–29 of the *SHPRH* gene and contains only 440 bases (Li et al., 2019) (Figure 3). According to current research, *SHPRH* plays a tumor suppressing function in cancer (Sood et al., 2003). By using the circBase database (www.circbase.org, accessed on 29 December 2022.), 25 human circRNAs were found to be generated from *SHPRH*, but only hsa_circ_0001649 has been studied. Some studies have confirmed that circ-SHPRH is under expressed in many kinds of cancer, such as gastric cancer, glioma, and cholangiocarcinoma (Li et al., 2017; Zhang M. et al., 2018; Xu et al., 2018). Although circ-SHPRH is classified as a noncoding RNA because of its highly conserved structure, it has been found to be capable of translation in recent years. SHPRH-146aa, a protein encoded by circ-SHPRH, can protect *SHPRH* from degradation by the ubiquitin‒proteasome and reduce the malignant behavior of glioma cells *in vivo* and *in vitro* (Zhang M. et al., 2018). In addition, current research has discovered its role in cancer diagnosis and prognosis, indicating that it may be a tumor marker (Zhang X. et al., 2018; Jiang et al., 2018; Liu et al., 2018). This article reviews recent progress on the expression patterns and roles of circ-SHPRH and expands on our understanding of the functions and mechanisms of circ-SHPRH in multiple malignancies.

## 2 Methods and results of the publication search

We retrieved 113 research papers from the PubMed, Web of Science, and Embase databases using the keywords “circSHPRH” or “hsa_circ_0001649” or “circular RNA SHPRH”. The publication search was updated to 24 December 2022. After excluding 54 duplicate publications, 59 studies were included for further screening. After screening titles, abstracts, and full texts, 18 research papers were included in the scope of this review, and 11 papers were selected for meta-analysis (Qin et al., 2016; Li et al., 2017; Zhang M. et al., 2018; Wang Y. et al., 2018; Zhang X. et al., 2018; Ji et al., 2018; Jiang et al., 2018; Liu et al., 2018; Xing et al., 2018; Xu et al., 2018; Zuo et al., 2022).

## 3 Circ-SHPRH in malignant tumors

### 3.1 Non-small-cell lung cancer (NSCLC)

Some researchers suggest that the number of lung cancer cases and deaths is on the rise globally (Bade and Dela Cruz, 2020). According to the morphology of tumor cells, lung cancer can mainly be divided into small cell lung cancer (SCLC) and NSCLC. Most lung cancer cases are NSCLC (85%), so NSCLC is currently the most familiar type of lung cancer (Siegel et al., 2013). Liu et al. found that the level of circ-SHPRH was decreased in NSCLC tissues and cells, and circ-SHPRH expression was negatively correlated with the progression of NSCLC (Liu et al., 2018). Through further experimental analysis, they found that patients with higher circ-SHPRH levels had longer overall survival than patients with lower circ-SHPRH levels. Moreover, multivariate Cox regression analysis found that circ-SHPRH was an independent prognostic biomarker in NSCLC patients (*p* = 0.031). A study of the effect of circ-SHPRH on the function of NSCLC cells showed that upregulated circ-SHPRH expression suppressed the proliferation and metastasis of NSCLC. In a further study of the mechanism of circ-SHPRH in NSCLC, circ-SHPRH was found to inhibit the proliferation, migration, and invasion of NSCLC by directly sponging miR-331-3p and miR-338-5p. These results suggest that the circ-SHPRH/miR-331-3p/miR-338-5p signaling pathway is a potential therapeutic target, but the downstream target genes of this pathway remain unexplored.

### 3.2 Hepatocellular carcinoma (HCC)

Liver cancer ranks third among the cancers that kill men and is sixth among the top ten most common cancers in the world (Sung et al., 2021). Liver cancer can be divided into two main categories: HCC (accounting for 75%–85%) and intrahepatic cholangiocarcinoma (accounting for 10%–15%). Some HCC patients develop symptoms late, making an early diagnosis difficult to obtain, and these patients have higher rates of metastasis and recurrence, resulting in a poor 5-year survival rate (Wang M. et al., 2018). Hence, finding new biomarkers is essential to improve the prognosis and treatment of HCC. With in-depth research on circRNA, new ideas have been provided for the early diagnosis and treatment of tumors. Circ-SHPRH has been found to have antitumor effects in HCC(Qin et al., 2016; Zhang X. et al., 2018; Su et al., 2019). Qin et al. found that circ-SHPRH is downregulated in HCC compared with normal liver (Qin et al., 2016). Circ-SHPRH expression reduction is associated with adverse HCC prognosis. Furthermore, the lower the circ-SHPRH expression in the tumor, the larger the tumor volume, which suggests that circ-SHPRH is related to tumor growth. In addition, ROC analysis showed that the area under the ROC curve for circ-SHPRH was 0.63, and its specificity and sensitivity were 0.81 and 0.69; thus, circ-SHPRH may be a potential biomarker for HCC diagnosis. In terms of function, Su et al. detected that overexpression of circ-SHPRH suppressed the proliferation and migration of HCC. Mechanistically, circ-SHPRH regulates the function of HCC cells by sponging miR-4688, miR-612, and miR-127-5p (Su et al., 2019). Similarly, Zhang et al. discovered that overexpression of circ-SHPRH suppressed the proliferation, migration, and invasion of HCC cells. Moreover, Cox multivariate analysis also suggested that circ-SHPRH is a new independent prognostic factor in HCC patients, but it was not associated with clinical characteristics, such as age, TNM stage and lymphatic metastasis (Zhang X. et al., 2018). In addition, overexpression of circ-SHPRH caused increased expression of caspase-3 and caspase-9 in hepatoma cells, further enhancing apoptosis (Sun et al., 2022). The results indicate that circ-SHPRH regulates the progression of HCC and is a potential treatment target for HCC.

### 3.3 Cholangiocarcinoma (CCA)

Cholangiocarcinoma (CCA) is a malignant tumor that occurs in the bile duct system, and its incidence is on the rise worldwide. Currently, it accounts for approximately 15% of all primary hepatobiliary malignancies, second only to hepatocellular carcinoma (Bergquist and von Seth, 2015). CCA is highly invasive and the therapeutic effects of chemotherapy and radiotherapy are poor, so the prognosis of CCA is also poor. Its overall incidence has gradually increased in the past few decades (Saha et al., 2016). Through qRT–PCR experiments, Xu et al. confirmed that circ-SHPRH expression was abnormally decreased in CCA tumor tissues (Xu et al., 2018). Fisher’s exact test results show that the level of circ-SHPRH is closely related to the volume and degree of differentiation of the tumor. Functionally, circ-SHPRH can suppress the proliferation, invasion, and migration of CCA cells and induce apoptosis of CCA cells. The above findings suggest that circ-SHPRH has potential value as a clinical diagnostic and prognostic predictor of CCA.

### 3.4 Pancreatic ductal adenocarcinoma (PDAC)

In recent years, the incidence of pancreatic ductal adenocarcinoma (PDAC) has been increasing, and its 5-year survival rate is only approximately 9%, making it a deadly malignant tumor (Siegel et al., 2020). Due to the lack of effective screening methods, many patients have been diagnosed with advanced cancer, so the prognosis of these patients is extremely poor (Stathis and Moore, 2010; Koch et al., 2020). Jiang et al. confirmed that circ-SHPRH expression was abnormally decreased in PDAC cells through qRT–PCR experiments (Jiang et al., 2018). In terms of clinicopathological features, Fisher’s exact test analysis showed that the level of circ-SHPRH was lower in poorly differentiated tumors (*p* = 0.018). Kaplan–Meier survival curve analysis found that patients with higher circ-SHPRH expression had better 5-year overall survival than patients with lower circ-SHPRH expression (*p* = 0.002). The level of circ-SHPRH was positively associated with the 5-year overall survival rate of patients. In addition, Cox regression analysis suggested that the level of circ-SHPRH was an independent prognostic factor for PDAC (*p* = 0.039). In further exploration of the role of circ-SHPRH in cell function, upregulation of circ-SHPRH was discovered to suppress the proliferation and colony formation of PDAC cells. Furthermore, circ-SHPRH promoted the apoptosis of PDAC cells by activating caspase-9 and caspase-3. Caspase-9 and caspase-3 can promote apoptosis and play important roles in cell apoptosis (Brentnall et al., 2013). In summary, circ-SHPRH inhibits PDAC growth and promotes apoptosis, so it may become a new PDAC therapeutic target in the future.

### 3.5 Gastric cancer (GC)

Gastric cancer (GC) is one of the most common malignant cancers in the world, and the incidence and mortality of gastric cancer rank fifth and fourth, respectively (Machlowska et al., 2020). Gastric cancer has a higher degree of malignancy and a poor prognosis, and many patients are diagnosed with advanced gastric cancer (Sung et al., 2021). A study by Li et al. discovered that the level of circ-SHPRH in gastric cancer tissues was significantly reduced compared with that in normal tissues (Li et al., 2017). By comparing the level of circ-SHPRH in serum samples of GC patients before and after surgery, the expression of circ-SHPRH in the serum of GC patients after surgery was found to be significantly increased. ROC curve analysis indicated that circ-SHPRH has high accuracy, specificity, and sensitivity, and can be a biomarker for GC. In addition, researchers discovered that the level of circ-SHPRH was correlated with the pathological differentiation of GC. This result suggests that circ-SHPRH has the potential to be used as a novel biomarker for GC. A study by Sun et al. also proved that circ-SHPRH is downregulated in GC (Sun et al., 2020). In addition, they found that upregulation of circ-SHPRH suppressed the proliferation, migration, and invasion of GC cells. It can also promote the apoptosis of GC cells. At present, studies have found that miR-20a expression is higher in GC and can promote the growth of GC cells (Jafarzadeh-Samani et al., 2017). Mechanistically, researchers found that circ-SHPRH further inhibited the downstream ERK and Wnt/β-catenin pathways, mainly by inhibiting miR-20a. Overexpression of miR-20a significantly reversed these effects. These data suggest that circ-SHPRH inhibits the ERK and Wnt/β-catenin pathways by downregulating the expression of miR-20a, thereby achieving a tumor suppressor effect (Huang et al., 2017; Yang et al., 2017).

### 3.6 Colorectal cancer (CRC)

Approximately 900,000 people worldwide die from colorectal cancer every year, often at an advanced stage after diagnosis (Sung et al., 2021). The prognosis of CRC patients remains poor due to postoperative recurrence and metastasis. Therefore, finding new biomarkers is essential to improve the prognosis and treatment of CRC. Ji et al. confirmed that the level of circ-SHPRH in colorectal cancer tissues was significantly lower than that in the corresponding normal tissues. In addition, by comparing the levels of circ-SHPRH in the serum of CRC patients before and after surgery, they discovered that the level of circ-SHPRH was noticeably increased after surgery. In addition, they discovered that the higher the level of circ-SHPRH, the better the pathological differentiation of colon cancer. This result indicated that the level of circ-SHPRH was correlated with pathological differentiation (*p* = 0.037). The results of the ROC curve analysis showed that circ-SHPRH has potential diagnostic value in CRC (Ji et al., 2018). These results indicate that circ-SHPRH can be used as a new biomarker for specific and sensitive CRC detection.

### 3.7 Bladder cancer (BC)

Bladder cancer (BC) is a relatively common malignant tumor of the urinary system, with more than 500,000 new cases worldwide every year (Lenis et al., 2020; Richters et al., 2020). Zuo et al. found that the level of circ-SHPRH in BC tissues and cell lines was significantly decreased and low expression of circ-SHPRH was associated with high grade, advanced pathological T stage and lymph node metastasis of BC. It has been reported that silencing of circ-SHPRH significantly enhances the proliferation, migration, and invasion of BC cells. Overexpression of circ-SHPRH inhibits tumor growth *in vivo*. Elevated circ-SHPRH upregulated the expression of a downstream gene (*BARX2*) by adsorbing miR-942 and effectively suppressed the proliferation, invasion, and migration of BC cells (Zuo et al., 2022). In conclusion, targeting circ-SHPRH to explore new therapeutic methods for BC has good application prospects.

### 3.8 Osteosarcoma (OS)

Osteosarcoma is a malignant tumor originating from mesenchymal tissue that mainly occurs in adolescents and has the characteristics of high malignancy and pulmonary metastasis (Bozorgi and Sabouri, 2021). Sun et al. found that upregulation of circ-SHPRH can inhibit the proliferation of OS cells and induce apoptosis. By qRT–PCR, they found that circ-SHPRH was dramatically reduced in OS compared with normal tissues (Sun and Zhu, 2020). Mechanistically, they found that there is a potential regulatory relationship between circ-SHPRH and miR-338-5p, miR-942, and miR-647. Further study confirmed that circ-SHPRH can combine with miR-338-5p, miR-647, and miR-942 to act as a molecular sponge, thereby inhibiting OS proliferation and inducing OS apoptosis (Liu et al., 2017; Long et al., 2018; Zhang et al., 2019). The STAT signaling pathway plays a significant role in cancer cell proliferation and differentiation and participates in tumor cell recognition and tumor-driven immune escape (Quesnelle et al., 2007; Roberts et al., 2007). Among the *STATs*, *STAT3*, and *STAT5* play a major role in cancer by mediating the processes of tumor generation and development and regulating downstream gene expression (Linher-Melville and Singh, 2017). circ-SHPRH can further inhibit the STAT pathway by combining with three miRNAs (miR-338-5p, miR-647, and miR-942).

### 3.9 Glioma

Glioma is a malignant tumor of the central nervous system. Because of its strong invasion and high recurrence rate, a surgical cure is difficult to achieve (Van Meir et al., 2010; Gittleman et al., 2018). Wang et al. discovered that circ-SHPRH was generally downregulated in glioma specimens (Wang Y. et al., 2018). They found that downregulated circ-SHPRH was related to larger tumor size and advanced grade. The study also found that the upregulation of circ-SHPRH can suppress the growth and cloning ability of glioma cells and promote the apoptosis of glioma cells. Bcl-2 and Bax regulate the release of cytochrome c and therefore play a central regulatory role in cell growth and apoptosis (Knudson et al., 1995; Evan and Littlewood, 1998). Researchers found that circ-SHPRH regulates apoptosis induced by the Bcl-2/caspase-3 pathway. In another study, Chen et al. examined 14 candidate circRNAs for analysis and finally screened three circRNAs (circFOXO3, circ0029426, and circ-SHPRH) whose expression in the serum of patients was much higher than that of healthy volunteers (Chen et al., 2020). The results showed that circ-SHPRH is a tumor suppressor in glioma and a possible biomarker for this type of cancer. In another study, Zhang et al. found that the circ-SHPRH encodes a new protein called SHPRH-146aa (Zhang M. et al., 2018). Compared with normal tissues, the expression levels of circ-SHPRH and SHPRH-146aa were both downregulated in gliomas. Overexpression of SHPRH-146aa can reduce the malignant behavior and tumorigenicity of glioma *in vitro* and *in vivo*. Mechanistically, SHPRH-146aa can protect *SHPRH* from degradation by the ubiquitin–proteasome and promote the ubiquitination of proliferating cell nuclear antigen (PCNA). These results suggest that SHPRH-146aa, encoded by circ-SHPRH, is a cancer suppressor in glioma.

### 3.10 Retinoblastoma (RB)

Retinoblastoma (RB) is a rare pediatric cancer, but it is the most common intraocular malignancy in adolescents. There are approximately 9000 newly diagnosed patients worldwide each year. In less developed regions, the mortality rate of retinoblastoma is approximately 70% (Dimaras et al., 2012). Through qRT–PCR detection, Xing et al. discovered that circ-SHPRH was reduced in RB tissue samples compared to normal tissue (Xing et al., 2018). After upregulation of circ-SHPRH, the proliferative capacity of RB cells was significantly reduced, and the proportion of apoptotic cells was significantly increased. Further exploration of the tumor suppressor mechanism showed that circ-SHPRH inhibited RB proliferation and promoted RB apoptosis by regulating the AKT/mTOR signaling pathway. The results of animal experiments showed that the growth of xenografts in the circ-SHPRH overexpression group was obviously slower than that in the blank group. Furthermore, Fisher’s exact test showed that circ-SHPRH was significantly related to cancer volume (*p* = 0.017) and advanced intraocular international retinoblastoma classify (IIRC) stage (*p* = 0.01). Kaplan–Meier survival analysis showed that the 5-year survival rate of patients with downregulated circ-SHPRH was poor (*p* = 0.005). Furthermore, Cox regression analysis showed that circ-SHPRH may be an independent prognostic indicator for RB (*p* = 0.023). In conclusion, enhancing the role of circ-SHPRH in RB may become a new therapeutic strategy.

### 3.11 Oral squamous cell carcinoma (OSCC)

Oral squamous cell carcinoma (OSCC) is one of the most common tumors of the head and neck, ranking eighth among the most common tumors in the world (Bouaoud et al., 2022). OSCC is the most common oral cancer with a poor prognosis and a high mortality rate. Tobacco, alcohol, and genetic alterations are the main factors. In addition, patients with secondary tumors have a lower survival rate because of the higher rate of secondary tumor formation compared with other tumors (Chamoli et al., 2021). Hung et al. found that the expression of circ-SHPRH was downregulated in OSCC tissues and plasma samples (Hung et al., 2022). The expression of circ-SHPRH in advanced OSCC patients was significantly lower than that in healthy controls. In addition, the expression of circ-SHPRH in the plasma of patients with recurrent OSCC was significantly lower than that of patients without recurrent OSCC. Patients with reduced plasma circ-SHPRH levels had a higher risk of early tumor recurrence and poor prognosis. These results suggested that circ-SHPRH, as a biomarker in plasma, has a high value in predicting recurrent OSCC. There is also great potential for risk classification and improved treatment strategies in OSCC patients.

## 4 Meta-analysis

### 4.1 Methods

#### 4.1.1 Inclusion and exclusion criteria

The inclusion criteria were as follows: (1) the expression level of hsa_circ_0001649 was detected in any type of human cancer; (2) studies in which patients were stratified by the expression levels of hsa_circ_0001649; (3) the association between hsa_circ_0001649 expression and OS and TNM stage was studied.

The exclusion criteria were as follows: (1) duplicate publications; (2) reviews, case reports, or letters; (3) studies that only investigated the biological function of hsa_circ_0001649.

#### 4.1.2 Data extraction

Two authors (Hong Xiong and Gaozhen Huang) extracted the data from the identified publications independently. The following information was extracted from each publication: name of the first author, publication year, country, cancer type, number of cases, detection method, survival outcome, follow-up time, etc.

#### 4.1.3 Quality assessment

The Newcastle‒Ottawa Score (NOS) quality assessment system was used to determine the quality of the enrolled studies (Stang, 2010). Enrolled studies were scored based on case definition, representation of cases, selection restrictions, definition of controls, comparability of cases and controls, determination of exposure, identical determination methods for cases and controls, and nonresponse rates. Studies with a score ≥6 were considered high quality.

#### 4.1.4 Statistical analysis

The results were visualized using Meta-Disc version 1.4 (the Unit of Clinical Biostatistics team of the Ramón y Cajal Hospital, Madrid, Spain), STATA version 17SE (Stata Corporation, College Station, TX, United States), and Review Manager version 5.3 (Copenhagen: the Nordic Cochrane Centre, the Cochrane Collaboration, 2014). Heterogeneity tests were performed by I-squared statistics. We analyzed the data using a fixed-effects model by default and switched to a random-effects model if I-squared was >50%. We determined that there was significant heterogeneity among the included studies when the *p*-value was <0.05; otherwise, there was no significant heterogeneity. For prognostic meta-analysis, pooled OR, as well as 95% CI, was used to describe the prognostic value of hsa_circ_0001649 expression. The potential publication bias was estimated by using Egger’s funnel plot. We determined that there was no publication bias if the *p*-value was >0.1 for Egger’s test.

## 5 Results

### 5.1 Characteristics of the enrolled studies

**FIGURE 4:**
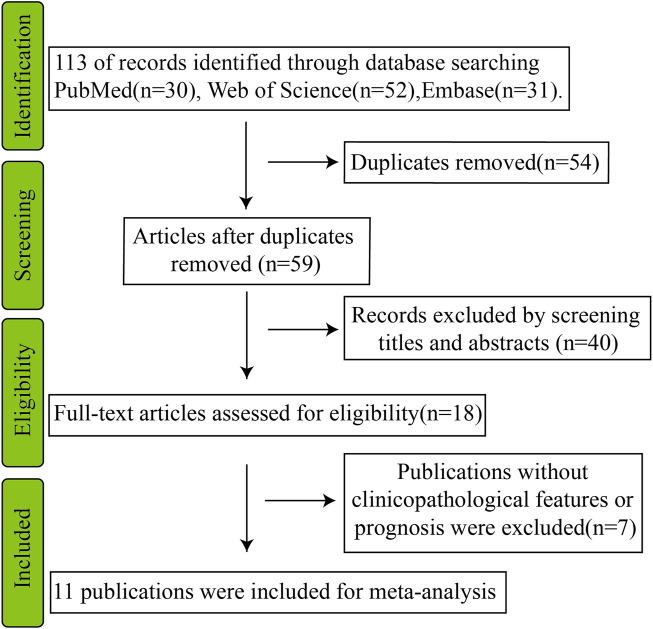
Methods and results of the publication search.

A flow chart of screening eligible articles for the meta-analysis is shown in Figure 4. The 11 included studies were published between 2016 and 2022. Three eligible published studies about circ-SHPRH were enrolled based on their tumor diagnosis aspect (Qin et al., 2016; Li et al., 2017; Ji et al., 2018), 7 eligible published studies were related to overall survival (OS) (Zhang M. et al., 2018; Wang Y. et al., 2018; Zhang X. et al., 2018; Jiang et al., 2018; Liu et al., 2018; Xing et al., 2018; Xu et al., 2018; Zuo et al., 2022), and 3 eligible published studies were related to tumor grade (Jiang et al., 2018; Liu et al., 2018; Xu et al., 2018). Expression of circ-SHPRH was measured using quantitative real-time polymerase chain reaction (RT-qPCR).

### 5.2 Potential diagnostic value of Circ-SHPRH in solid tumors

**FIGURE 5:**
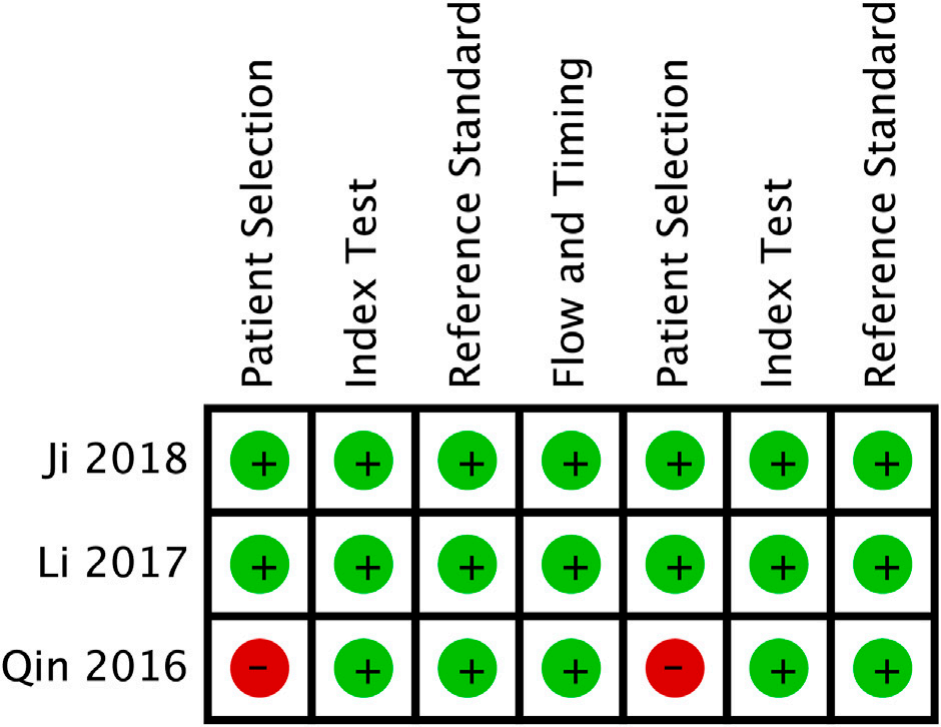
Quality assessment of eligible studies.

**FIGURE 6:**
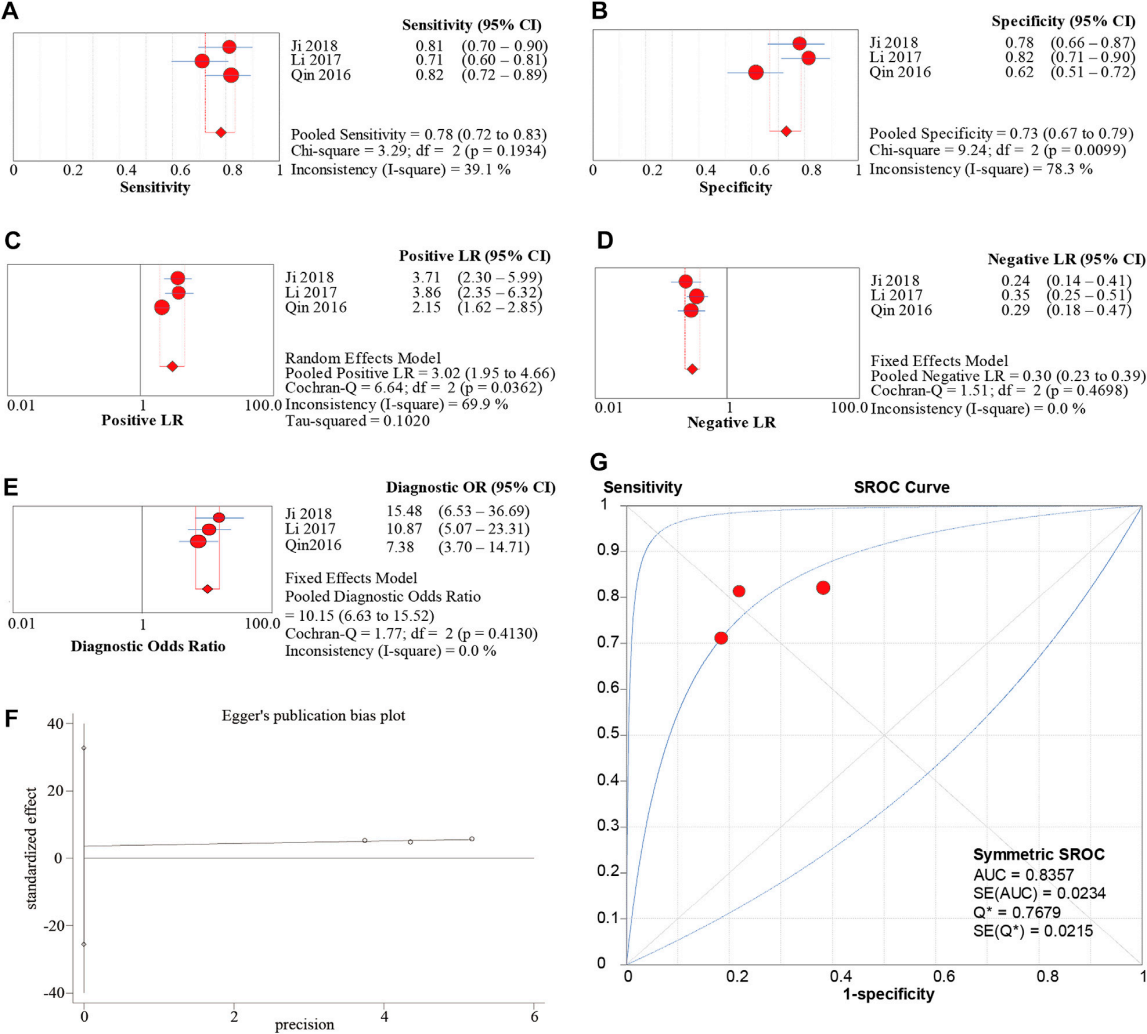
**(A–D)** Sen, Spe, PLR, NLR of forest plot of diagnostic accuracy index for circ-SHPRH in solid cancer. **(E)** DOR (diagnostic odds ratio) of circ-SHPRH in solid cancer. **(F)** Egger’s publication bias plot. **(G)** SROC (summary receiver operator characteristic curve) of circ-SHPRH in solid cancer.

A total of 3 publications involving 229 cases were included in the diagnostic meta-analysis. The main characteristics of all included studies are shown in Table 1. The quality assessment results of the diagnostic studies are shown in Figure 5. The meta-analysis of the sensitivity and specificity of circ-SHPRH showed that the combined study had a sensitivity and specificity of 0.78 (0.72–0.83) and 0.73 (0.67–0.79), respectively, and a PLR and an NLR of 3.02 (1.95–4.66) and 0.30 (0.23–0.39), respectively, which are more important values than the first two indicators (Figures 6A–D). Due to the high heterogeneity of the specificity and PLR, we switched to a random effects model for analysis. The DOR value was 10.15 (6.63–13.52) (Figure 6E). The corresponding area under the SROC curve (AUC) was calculated to be 0.8357 (Q* = 0.7679), revealing moderate diagnostic accuracy (Figure 6G). Egger’s publication bias plot was conducted to evaluate publication bias. The shape of the funnel plots showed symmetry for all included studies and the *p*-value of Egger’s test was 0.296 (Figure 6F).

**TABLE 1:** Characteristics of the studies included in the present meta-analysis.

Study	Year	Region	Cancer type	Sample size		Detection method	Cutoff value	AUC	Sen	Spe	TP	FP	FN	TN
				Cases	Control									
Ji	2018	China	CRC	64	64	qPCR	0.283467282	0.852	0.806	0.787	52	14	12	50
Li	2017	China	GC	76	76	qPCR	0.34626743	0.83	0.738	0.794	54	14	22	62
Qin	2016	China	HCC	89	89	qPCR	0.42097195	0.715	0.774	0.682	73	34	16	55

Abbreviations: CRC, colorectal cancer; GC, gastric cancer; HCC, hepatocellular carcinoma; qPCR, quantitative real-time polymerase chain reaction; AUC, area under the curve; Sen, sensitivity; Spe, specificity; TP, true positive; FP, false positive; FN, false negative; TN, true negative.

### 5.3 Association between Circ-SHPRH expression and the prognosis of patients with malignant tumors and publication bias

**FIGURE 7:**
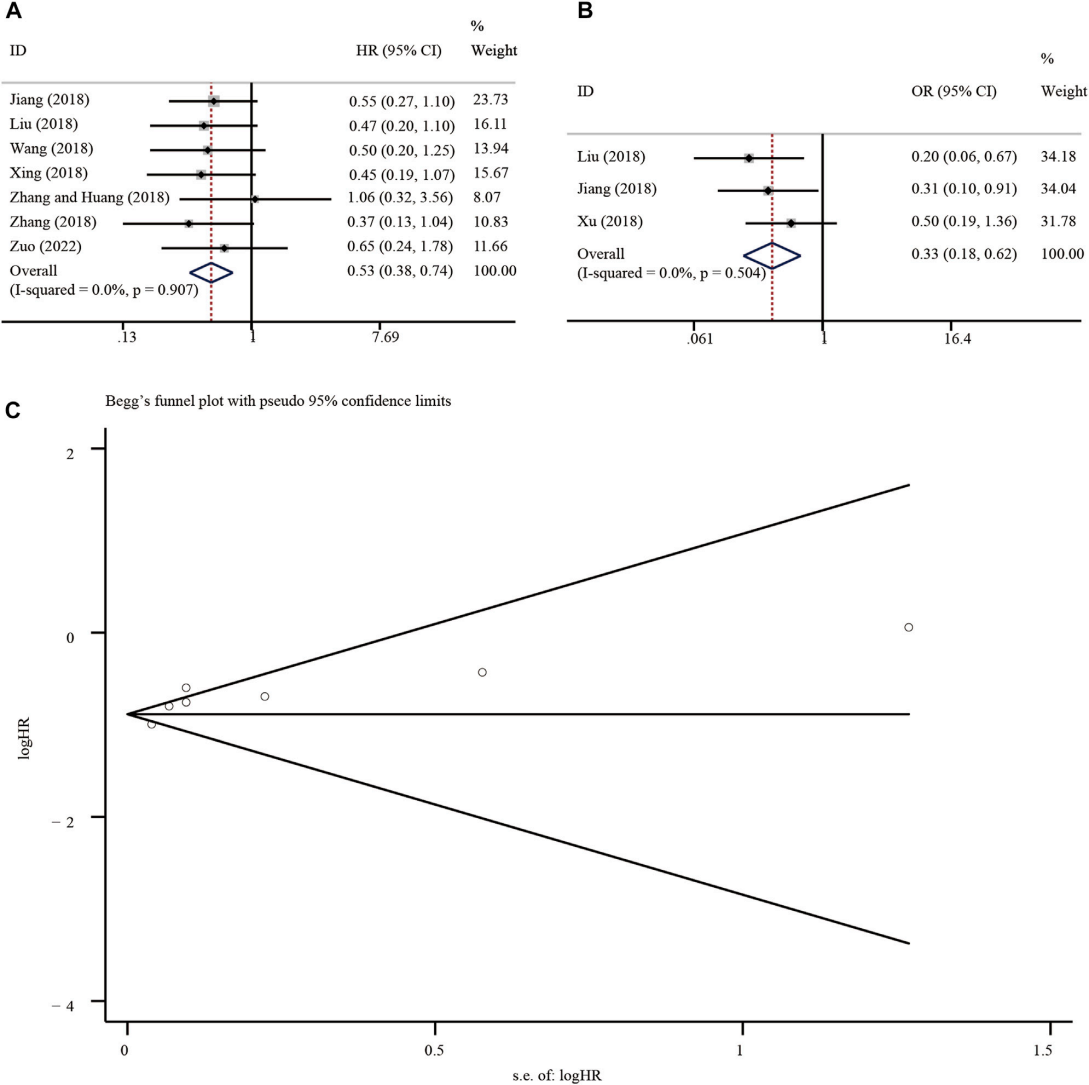
**(A, B)** Forest plot of the association of circ-SHPRH expression with OS and TNM stage. **(C)** Begg’s funnel plot.

Meta-analysis of 7 eligible studies, involving 434 patients, showed better OS in malignancies with high circ-SHPRH expression (HR = 0.53, 95% CI 0.38–0.74, I-squared = 0.0%, *p*-value <0.05) (Figure 7A) (Table 2). To further understand the association between circ-SHPRH expression and TNM stage, a meta-analysis of 3 eligible studies, involving 187 patients, was performed. The results showed that patients with higher circ-SHPRH expression had lower TNM stage (HR = 0.33, 95% CI 0.18–0.62, I-squared = 0.0%, *p*-value = 0.001) (Figure 7B). The potential publication bias was estimated by using Begg’s funnel plot. As shown in Figure 7C, the Begg’s funnel plot showed symmetry, the *p*-value = 0.649.

**TABLE 2:** Main characteristics and quality assessment of eligible studies for prognosis.

Study	Year	Region	Cancer type	Case	Detection method	Follow up	Survival	Quality (NOS)
Jiang	2018	China	PDAC	58	qPCR	≥60	OS, TNM	7
Liu	2018	China	NSCLC	53	qPCR	≥60	OS, TNM	7
Wang	2018	China	Glioma	64	qPCR	≥60	OS	7
Zhang	2018	China	Glioma	60	qPCR	≥25	OS	7
Xing	2018	China	RB	60	qPCR	≥60	OS	7
Zhang	2018	China	HCC	77	qPCR	≥50	OS	6
Zuo	2022	China	BCa	62	qPCR	≥80	OS	6
Xu	2018	China	CCA	76	qPCR	NA	TNM	5

Abbreviations: PDAC, pancreatic ductal adenocarcinoma; NSCLC, non-small-cell lung cancer; RB, retinoblastoma; HCC, hepatocellular carcinoma; BCa, bladder cancer; CCA, cholangiocarcinoma; OS, overall survival.

## 6 Conclusions and prospects

**FIGURE 8:**
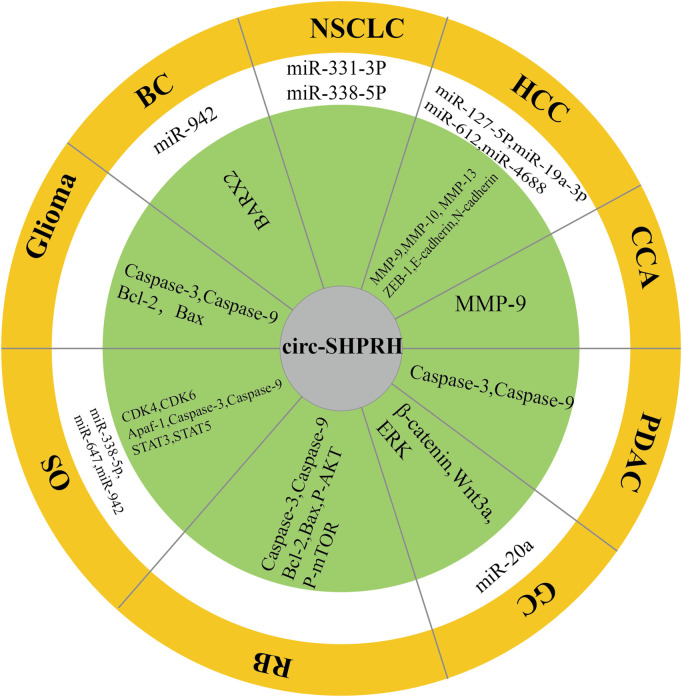
Schematic illustration showing the molecular mechanisms of circ-SHPRH in multiple cancers. The white area represents miRNAs that can be sponged by circ-SHPRH. The green area represents targeted genes and proteins associated with circ-SHPRH.

In this review, we introduce many novel features of circ-SHPRH to further understand the role of circ-SHPRH in regulating gene expression in tumorigenesis and tumor progression. Functionally, circ-SHPRH has been shown to be a tumor suppressor that inhibits tumor progression in a variety of cancers. Mechanismly, circ-SHPRH has been shown to be a molecular sponge that can competitively “take up miRNAs” and perform different functions by combing with them (Figure 8). Numerous studies have revealed correlations between circ-SHPRH and tumor clinicopathological features (Table 3). Meta-analysis showed that patients with high expression of circ-SHPRH had better OS and lower TNM stage. In addition, circ-SHPRH may also become a new tumor marker candidate, providing a new target for the diagnosis and prognosis of different types of cancer. However, current findings on the role of circ-SHPRH are limited. For example, the sample sizes of many studies were small. Although several studies have revealed the correlation of tumor clinicopathological features with circ-SHPRH, retrospective and prospective studies of large patient samples are still needed to provide more evidence to support the correlation of circ-SHPRH with specific cancers. Current studies have mainly focused on the interaction of circ-SHPRH with miRNAs in tumors, but this does not represent all functions of circ-SHPRH. It has been reported that circ-SHPRH also encodes a protein called SHPRH-146aa in glioma. In addition, we used the circFunBase online database (https://bis.zju.edu.cn/CircFunBase/, accessed on 29 December 2022.) to predict potential RNA-binding proteins that can bind to circ-SHPRH. The results showed that EIF4A3 and AGO2 could bind to circ-SHPRH and participate in RNA‒protein interactions. Therefore, it is very important to study other novel functions and mechanisms of circ-SHPRH. In addition, in view of the current research on circ-SHPRH in tumors, the next research direction will be how to translate our knowledge of circ-SHPRH to develop clinically applicable treatment methods, such as targeted drugs for circ-SHPRH. With the emergence of *in vitro* synthesis of circRNA technology (Qu et al., 2022), synthetic drugs targeting circ-SHPRH may become a treatment choice for certain cancers in the future.

**TABLE 3:** Functional features of circ-SHPRH in various human cancers.

Cancer types	Expression	Function	Clinicopathological feature	Clinical relevance	Sample size	Ref
NSCLC	Down	Proliferation, Migration	TNM stage	Prognosis	53	Liu et al., 2018
		Invasion	lymph node metastasis			
HCC	Down	Migration	Tumor size, tumor embolus	Diagnostic	89	Qin et al., 2016
	Down	Proliferation, Migration	OS	Prognosis	77	Zhang et al., 2018a
		Invasion, Apoptosis				
	Down	Proliferation	Grade of differentiation	—	84	Su et al., 2019
			tumor satellite	—		
	Down	Proliferation, Migration	—	—	—	Sun et al., 2022
		Invasion, Apoptosis				
CCA	Down	Migration, Invasion, Apoptosis	Tumor size	Prognosis	76	Xu et al., 2018
			grade of differentiation			
PDAC	Down	Proliferation, Apoptosis	Tumor stage	Prognosis	58	Jiang et al., 2018
			grade of differentiation, OS			
GC	Down	Proliferation, Migration	—	—	25	Sun et al., 2020
		Invasion, Apoptosis				
	Down	—	Pathological differentiation	Diagnostic	76	Li et al., 2017
CRC	Down	—	Pathological differentiation	Diagnostic	64	Ji et al., 2018
BC	Down	Proliferation, Migration, Invasion	—	Prognosis	62	Zuo et al., 2022
OS	Down	Proliferation, Apoptosis	—	—	26	Sun et al., 2020
Glioma	Down	Proliferation, Apoptosis	Tumor size, WHO grade	Prognosis	64	Wang et al., 2018a
	Down	—	—	—	100	Chen et al., 2020
	Down	Proliferation	OS	Prognosis	60	Zhang et al., 2018b
RB	Down	Proliferation, Apoptosis	Tumor size, IIRC stage, OS	Prognosis	60	Xing et al., 2018
OSCC	Down	—	Recurrence	—	66	Hung et al., 2022
